# Increased risk of rehospitalisation and death in older hospital patients at risk of malnutrition: A cohort study

**DOI:** 10.1016/j.jnha.2024.100455

**Published:** 2024-12-19

**Authors:** Kristin I. Folven, Eva Biringer, Roy M. Nilsen, Anne Marie Beck, Kari Sygnestveit, Eli Skeie, Øystein Hetlevik, Randi J. Tangvik

**Affiliations:** a Department of Research and Innovation, Fonna Hospital Trust, P.O. Box 2170, NO-5504 Haugesund, Norway; b Centre for Nutrition, Department of Clinical Medicine, University of Bergen, P.O. Box 7804, NO-5020 Bergen, Norway; c Faculty of Health and Social Sciences, Western Norway University of Applied Sciences, P.O. Box 7030, NO-5020 Bergen, Norway; d Unit for Dieticians and Nutrition Research, Herlev and Gentofte University Hospital, DK-2730 Herlev, Denmark; e Department of Research and Development, Haukeland University Hospital, Bergen, Norway; f Norwegian National Advisory Unit on Disease Related Undernutrition, Oslo University Hospital, Oslo, Norway; g Department of Health and Social Services, Kvam Municipality, Norheimsund, Norway; h Department of Global Public Health and Primary Care, Faculty of Medicine, University of Bergen, P.O. Box 7804, NO-5020 Bergen, Norway

**Keywords:** Nutritional status, Nutrition assessment, Aged, Hospitals, Health services, Mortality

## Abstract

**Objectives:**

The aim of the study was to investigate associations between risk of malnutrition and risk of rehospitalisation and death in older hospital patients, and whether the possible associations were modified by age, gender, comorbidity or Ambulatory Care Sensitive Conditions (ACSCs).

**Design:**

Prospective cohort study.

**Setting:**

Somatic hospital in Western Norway.

**Participants:**

9,768 hospital admissions for patients aged ≥65 years.

**Measurements:**

Information on the risk of malnutrition was based on nutritional risk screening data from 34 point prevalence surveys conducted between 2008 and 2018. Risk of malnutrition was assessed using Nutritional Risk Screening 2002 (NRS 2002) during the initial hospital admission. Outcomes and possible effect modifiers were obtained from the hospital’s patient administration systems. The short-term outcome was the length of initial hospital stay. Long-term outcomes included total number of days in hospital, number of hospital stays and risk of death within one year following nutritional risk screening. Statistical analysis involved negative binomial and Cox regression models with adjustment for age, sex and number of diagnoses at time of nutritional risk screening.

**Results:**

Overall, 34% of the patients were classified as being at risk of malnutrition. A higher proportion of the initial admissions were related to ACSCs for patients at risk of malnutrition than for those not at risk. Risk of malnutrition was associated with longer initial hospital stay (adjusted hazard ratio (95% confidence intervals) 1.31 (1.25, 1.37)), more days in hospital (adjusted risk ratio 1.25 (1.18, 1.32)) and a higher risk of having more than two hospital admissions the year following nutritional risk screening (adjusted risk ratio 1.16 (1.07, 1.26)). Patients at risk of malnutrition also had an increased risk of death within one year (adjusted hazard ratio 2.45 (2.25, 2.67)). All associations were more pronounced in the ‘65–69’ and ‘70–79’ age groups compared to the 80+ years age group, and in patients with fewer than four diagnoses compared to patients with four or more diagnoses. No significant interaction was detected between sex and risk of malnutrition with regard to patient outcomes.

**Conclusion:**

Older patients at risk of malnutrition have a higher risk of rehospitalisation and death during the first year after nutritional risk screening compared with those not at risk. Among patients at risk of malnutrition, the initial hospital admissions were more often due to ACSCs. The impact of the risk of malnutrition on outcomes appears stronger in patients aged 65–79 years and in patients with less comorbidity. These findings underline the importance of nutritional risk screening and subsequent nutritional support in all groups of older patients.

## Highlights

• Prospective cohort study in Norwegian somatic hospitals over 11 years.
• Nutritional risk screening of patient ≥65 years of age from 9,768 admissions.
• Risk of malnutrition is associated with increased hospitalisation and death.
• Stronger associations in patients aged 65–79 years and patients with <4 diagnoses.

## 1 Introduction

There is a high prevalence of malnutrition in older patients [1,2] and the occurrence is expected to rise even more due to the demographic change towards an older population [3]. Associations between malnutrition, lower functional level and increased risk of complications, such as pressure ulcers, falls and infections, in older patients are established [[4], [5], [6]].

Routine screening for the risk of malnutrition using validated nutritional risk screening tools is recommended for all older persons [7]. Risk of malnutrition is associated with short-term outcomes, such as in-hospital mortality and length of initial hospital stay in older patients [4,8]. Although a review reported only a few high-quality studies focused on older patients, long-term mortality also seems to be associated with the risk of malnutrition in this group [8]. A recent Danish study by Iversen et al. found a higher risk of readmissions within 180 days after hospital discharge for older patients at risk of malnutrition [9]. However, that study did not report days in hospital during the readmissions. From a healthcare service perspective, we need knowledge of how the risk of malnutrition in older patients affects both the risk of readmission to hospital and the total number of days spent in hospital an extended period after an initial discharge.

Older patients are also vulnerable to hospitalisation due to ambulatory care sensitive conditions (ACSCs) [10], which are defined as conditions in which sufficient primary care can reduce unnecessary hospital admissions [11,12]. Several ACSCs may have an association with nutritional status; however, to our knowledge, studies are lacking on the relationship between the risk of malnutrition and ACSCs in older patients.

In the present study, we aimed to investigate associations between the risk of malnutrition and the length of the initial hospital stay, the total number of days in hospital, the number of hospital admissions and the risk of death within one year after nutritional risk screening in older patients. Furthermore, we aimed to investigate whether any of these possible associations were modified by age, gender, comorbidity or ACSCs. Thus, the goal of the study was to provide a deeper understanding of whether malnutrition has greater consequences in some groups and where to place targeted interventions to reduce negative patient outcomes and healthcare costs.

## 2 Materials and methods

### 2.1 Data source

This study was based on surveillance data collected at Helse Bergen Hospital Trust, Norway, between 2008 and 2018 [13]. The data included hospitalised adult patients screened for nutritional risk using the Nutritional Risk Screening 2002 (NRS 2002) [14] in a total of 34 point prevalence surveys in somatic wards at Haukeland University Hospital. Patients who were pregnant or admitted for bariatric surgery were excluded. The surveillance data were further linked to data from the electronic patient administrative systems to obtain information about study outcomes (length of initial stay, number of days in hospital and number of hospital stays within one year and date of death), using the patients’ national identity number.

### 2.2 Study sample

**Fig. 1:**
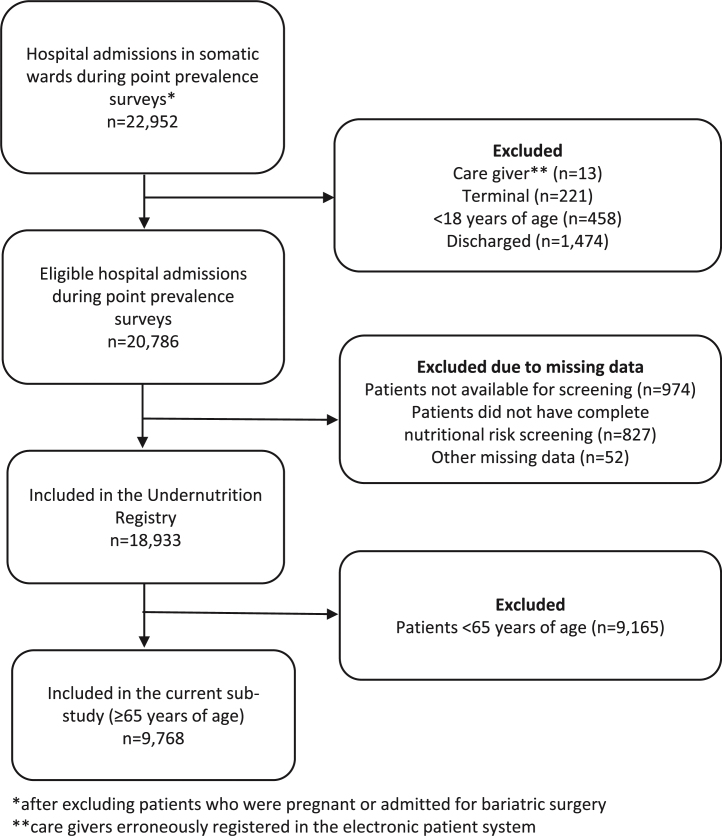
Flow chart: Inclusion of patients in the study.

A flow chart of the study sample is shown in Fig. 1. Patients in a terminal phase or less than 18 years of age were excluded before nutritional risk screening was performed. Some patients were unavailable for screening (e.g., out of ward for radiology procedures), and some were discharged before nutritional risk screening was completed in the wards (see Fig. 1 for details). Eleven percent of the patients had more than one hospital admission on a point prevalence survey date in the study period (range: 2–7 admissions).

The final study sample included 9,768 hospital admissions from 8,539 older patients aged ≥65 years. This represented 91% of all hospital admissions for patients eligible for nutritional risk screening (Fig. 1).

### 2.3 Risk of malnutrition

Patients were screened for risk of malnutrition using Nutritional Risk Screening 2002 (NRS 2002) [14]. The NRS 2002 includes four initial screening questions (yes/no) and a main screening section giving a nutritional risk score between 0 and 7 (see Table 1 for details on items and response options in the survey). The main screening was only performed if there was at least one affirmative response ‘yes’ in the initial screening. Patients who received a score of ≥3 in the main screening were classified as ‘at risk of malnutrition’. NRS 2002 was the screening tool used in daily nutritional practice in the hospitals during the years of the study. The NRS 2002 survey data were collected and registered by nurses, nurse assistants or physicians at the wards. More details on the data collection procedures can be found in reports by Tangvik et al. [15] and Skeie et al. [13].

**Table 1:** Questions and response options of the Norwegian version of NRS 2002^a^ in the point prevalence surveys.

Question/item	Response options
**NRS 2002 initial screening**	
Is BMI < 20.5?	Yes/No
Has the patient lost weight in recent weeks?^b^	Yes/No
Has the patient been eating less in the recent weeks?^b^	Yes/No
Is the patient severely ill?	Yes/No

**NRS 2002 main screening**	
Score for impaired nutritional status^c^	Absent = 0/Mild = 1/Moderate = 2/Severe = 3
Score for severity of disease	Absent = 0/Mild = 1/Moderate = 2/Severe = 3
Age ≥ 70 years	No = 0, Yes = 1
Total score (sum of the three main screening items)	0−7

NRS 2002 = Nutritional risk screening 2002, BMI = Body Mass Index.

a The early Norwegian version of the NRS 2002 had two minor deviations from the original version [14] and the revised Norwegian version [16] 1.

b ‘Has the patient lost weight/been eating less in *recent weeks*?’ versus ‘Has the patient lost weight within 3 months?’ and ‘Has the patient been eating less in the recent week?’ 2.

c The scores for impaired nutritional status were based on weight loss (%), food intake (% of normal intake) and BMI, thus did not include reduced general condition as a criterion.

### 2.4 Outcomes

The following study outcomes were derived from the electronic patient administration systems:

• Length of the initial hospital stay in days (i.e. number of days from the date of admission to the date of discharge for patients included in the prevalence surveys).
• Number of hospital admissions within one year following the nutritional risk screening date. This was dichotomised (0–2 and >2) to enable analysis of the risk for more than two hospital admissions within the first year after nutritional risk screening, as this was considered a relevant number of readmissions and gave two relatively balanced groups to compare statistically.
• Total number of days in hospital within one year following the nutritional risk screening date (i.e. aggregating all days in hospital for all hospital stays within one year, excluding the initial hospital admission).
• Date of death (i.e. time to death from the date of the nutritional risk screening in the initial hospital admission).

These four outcomes were chosen as they are indicators of health and health service use in hospital patients.

### 2.5 Other variables

We also used the electronic patient administration systems to obtain data on the patient’s age, sex, type of admission (acute, planned) and comorbidity (number of diagnoses). Diagnoses were classified using the International Statistical Classification of Diseases and Related Health Problems (ICD-10) and included one primary and up to nine secondary diagnoses at the initial hospital admission. The primary diagnosis was further used to define whether the initial hospital admission was due to an ambulatory care sensitive condition (ACSC) (see Supplementary Table S1 for the ICD-10 diagnoses that were included as ACSCs). The diagnoses were also used to determine the proportion of patients who had received a diagnosis related to malnutrition (i.e. ICD-10 codes E43, E44 or E46).

### 2.6 Statistics

All analyses were performed using Stata 17.0 (StataCorp, College Station, Texas, US). The associations of the risk of malnutrition with each of the four outcomes described above were assessed by applying log-binomial, negative binomial and Cox regression models. The patients’ nutritional risk status (‘at risk of malnutrition’ or ‘not at risk’) was entered as a binary categorical independent variable in the models, using ‘not at risk of malnutrition’ as the reference category. In our analysis of the length of initial stay, we instead calculated the hazard ratio (HR) for ‘at risk of malnutrition’ compared with patients ‘not at risk’. This was done to enable interpretation of the risk in the same direction as the other outcomes.

The statistical models used for investigating the associations of risk of malnutrition with each outcome were as follows:

• Length of initial hospital stay: Cox regression to estimate the HR and 95% confidence interval (CI) for discharge within 30 days as outcome. In this analysis, the length of stay was treated as a time-to-event variable, with discharge being the event of interest. Patients were censored at death or 30 days after the admission date.
• Total number of days in hospital: Negative binomial regression to estimate the risk ratio (RR) and 95% CI for having a greater number of days in hospital within one year following nutritional risk screening in the point prevalence survey.
• Number of hospital admissions: Log-binomial regression to estimate the RR and 95% CI of having more than two hospital admissions within one year following nutritional risk screening in the point prevalence survey.
• Risk of death: Cox regression to estimate the HR and 95% CI for death within 12 months as outcome (i.e. 365 days). HRs were also estimated for death within three and six months (i.e. 91 and 183 days).

Analyses were adjusted for patients’ age, sex, number of diagnoses (comorbidity), whether the admission was planned, and the year of the point prevalence survey. The year of the prevalence survey was operationalised as the number of years after the start of the study period (i.e. after 2008). The unit of analysis was hospital admission (i.e. patients were allowed to have more than one registration). Thus, to account for the clustering of hospital admissions within patients, 95% confidence intervals were estimated using robust standard errors.

To account for the risk of oversampling of patients with long-term initial stays in the point prevalence survey (i.e. length bias), we also incorporated individual sampling weights (i.e. maximum length of stay/individual length of stay) in the log-binomial and negative binomial regression models using the *pweight* function in Stata. This method of correction for length bias was not available for Cox regression models in Stata.

Finally, all the exposure-outcome associations were also investigated separately for men and women, for patients having <4 and ≥4 diagnoses, for four different age groups and for patients with initial admissions due to ACSCs and not due to ACSCs. Potential effect modification was evaluated using interaction analysis and the Wald test.

### 2.7 Ethics

The current study was approved by the Regional Committee for Medical and Health Research Ethics (REC North, Ref. No. 2018/1136). The approval included exemption from patient consent. A Data Protection Impact Assessment (DPIA) was performed by the Norwegian Centre for Research Data to ensure that data handling was in accordance with the General Data Protection Regulation (GDPR) of the European Union.

## 3 Results

### 3.1 Patient characteristics

The patient and nutritional characteristics, use of healthcare services and death in the total study sample (n = 9,768), for patients at risk of malnutrition (n = 3,278) and patients not at risk (n = 6,490), are presented in Table 2. A small proportion of the patients at risk of malnutrition (21.1%) had an ICD-10 diagnosis related to malnutrition.

**Table 2:** Patient characteristics, nutritional risk screening, hospitalisation and patient outcomes.

		Risk of malnutrition	
	Total screened	Yes	No
All admissions	9,768^a^	3,278^b^	6,490^b^
**Patient characteristics**			
Age, years (median, 25, 75 percentile)	76 (70, 82)	77 (71, 83)	75 (69, 82)
Sex, male (n, %)	4,971 (50.9)	1617 (49.3)	3,354 (51.7)
BMI, kg/m^2^ (mean ± SD)	25.0 ± 4.9	21.9 ± 4.8	26.5 ± 4.2
Number of diagnoses (n, %)			
1	966 (9.9)	157 (4.8)	809 (12.5)
2	1385 (14.2)	326 (10.0)	1,059 (16.3)
3	1635 (16.7)	503 (15.3)	1,132 (17.4)
≥4	5746 (58.8)	2275 (69.4)	3471 (53.5)
Acute admissions initially (n, %)	4646 (47.7)	1826 (55.9)	2820 (43.6)
Ambulatory care sensitive condition (ACSC)^c^ as cause of initial admission (n, %)	1,125 (11.5)	430 (13.1)	695 (10.7)

**Nutritional characteristics**			
NRS 2002 Initial screening (n, %)			
BMI <20.5 kg/m^2^? (yes)	1,619 (16.7)	1,585 (49.3)	34 (0.5)
Lost weight in recent weeks? (yes)	2309 (23.7)	1,730 (53.2)	579 (8.9)
Reduced dietary intake in recent weeks? (yes)	2819 (28.9)	2,182 (66.9)	637 (9.8)
Severely ill? (yes)	1229 (12.6)	859 (26.4)	370 (5.7)
Diagnosis related to malnutrition, ICD-10 codes E43, E44 or E46 (n, %)	866 (8.9)	693 (21.1)	173 (2.7)

**Outcome measures**			
Length of initial hospital admission, days (median, 25, 75 percentile)	9 (4, 17)	11 (6, 21)	8 (4, 15)
Days in hospital one year after nutritional risk screening (median, 25, 75 percentile)^d^	8 (3, 22)	12 (5, 27)	7 (2, 20)
Number of hospital admissions one year after nutritional risk screening (n, %)			
0	4,420 (45.3)	1303 (39.8)	3,117 (48.0)
1	1,976 (20.2)	686 (20.9)	1,290 (19.9)
2	1163 (11.9)	447 (13.6)	716 (11.0)
>2	2209 (22.6)	842 (25.7)	1367 (21.1)
Died within 12 months (n, %)	2,380 (24.4)	1,312 (40.0)	1,068 (16.5)

a 9,768 registrations in point prevalence surveys from 8,539 individual patients.

b At risk of malnutrition was defined as NRS 2002 score ≥3.

c Ambulatory care sensitive condition (ACSC): Primary diagnosis of dehydration, constipation, lower respiratory infection, urinary infection, gastroenteritis, fractures, anaemia, pressure ulcers or hospital admission due to problems related to care.

d Excluding initial hospital admission.

The proportion of patients initially admitted to hospital due to an ACSC was higher for patients at risk of malnutrition (13.1% (95% CI: 12.0, 14.3)) than for patients not at risk (10.7% (95% CI: 10.0, 11.5)). The percentage of acute admissions to hospital was 55.9% (95% CI: 54.0, 57.4) in patients at risk of malnutrition, and 43.6% (95% CI: 42.3, 44.7) in patients not at risk.

### 3.2 Risk of hospitalisation

Table 3 shows the effect estimates for the associations of risk of malnutrition with each of the four outcomes. Risk of malnutrition was associated with an increased HR of delayed discharge from hospital (adjusted HR (95%CI): 1.31 (1.25, 1.37)), compared to patients not at risk. We also found a greater risk of more days in hospital within one year following nutritional risk screening in patients at risk of malnutrition than in those not at risk (adjusted risk ratio (RR) (95% CI): 1.25 (1.18, 1.32)). The risk of having more than two hospital admissions was also higher in patients at risk of malnutrition than in those not at risk (adjusted RR (95% CI): 1.16 (1.07, 1.26)).

**Table 3:** Risk of hospitalisation and death in patients at risk of malnutrition versus not at risk.

Outcome	Model	Crude model	Adjusted model^a^
Length of initial hospital admission	Cox regression, HR (95% CI)	1.44 (1.38, 1.51)	1.31 (1.25, 1.37)^b^
Days in hospital one year after nutritional risk screening	Negative binomial regression, RR (95% CI)	1.33 (1.25, 1.41)	1.25 (1.18, 1.32)^c^
>2 hospital admissions one year after nutritional risk screening	Log-binomial regression, RR (95% CI)	1.22 (1.13, 1.32)	1.16 (1.07, 1.26)^d^
Risk of death within 12 months	Cox regression, HR (95% CI)	2.88 (2.65, 3.13)	2.45 (2.25, 2.67)^e^

HR = Hazard Ratio. RR = Risk Ratio. Reference group in the models was hospital admissions in which patients were not at risk of malnutrition except for length of hospital stay (measured as time to hospital discharge), where the HRs were inverted to enable interpretation of estimates in the same direction as in the other models.

a Analyses adjusted for age, sex, prevalence year, number of diagnoses and acute admissions. Valid n in adjusted analyses:

b n = 9,714.

c n = 9,729.

d n = 9,729.

e n = 9,722.

### 3.3 Risk of death

In total, 12% of the patients died within three months, 18% within six months and 24% within one year following nutritional risk screening. The proportion that died was higher in patients at risk of malnutrition than in patients not at risk at all three time points. The hazard rate for death within one year was nearly 2.5 times higher for patients at risk of malnutrition than for patients not at risk (adjusted HR (95% CI): 2.45 (2.25, 2.67)) (see Table 3). This increased risk was even more pronounced at three and six months after the nutritional risk screening (see Supplementary Table S3).

### 3.4 Possible effect modification by sex, age, comorbidity and ACSC

We found significant interactions between the risk of malnutrition and patients’ age for all four outcomes (Table 4). Investigation of the associations between the risk of malnutrition and outcome risks within each age group revealed a stronger association in the youngest age groups than in the oldest age groups.

**Table 4:** Risk of hospitalisation and death in patients at risk of malnutrition versus those not at risk stratified by age categories and comorbidity.

Age groups^a^				
Outcome		Crude model	Adjusted model^f^	p for interaction^h^
Length of initial hospital admission, HR (95% CI)^c^				0.041
	65–69 y	1.51 (1.35, 1.69)	1.24 (1.11, 1.40)	
	70–79 y	1.57 (1.47, 1.69)	1.38 (1.29, 1.49)	
	80–89 y	1.32 (1.22, 1.44)	1.25 (1.15, 1.36)	
	90+ y	1.24 (1.04, 1.48)	1.20 (1.01, 1.43)	
Days in hospital one year after nutritional risk screening, RR (95% CI)^d^				<0.001
	65–69 y	1.60 (1.40, 1.82)	1.42 (1.25, 1.62)	
	70–79 y	1.49 (1.36, 1.63)	1.39 (1,27, 1.52)	
	80–89 y	1.11 (1.01, 1.22)	1.07 (0.98, 1.17)	
	90+ y	0.90 (0.74, 1.09)	0.88 (0.73, 1.06)	
>2 hospital admissions one year after nutritional risk screening, RR (95% CI)^e^				<0.001
	65–69y	1.33 (1.13, 1.56)	1.15 (0.97, 1.36)	
	70–79 y	1.44 (1.29, 1.61)	1.30 (1.15, 1.47)	
	80–89 y	1.09 (0.94, 1.27)	1.06 (0.91, 1.24)	
	90+ y	0.48 (0.31, 0.76)	0.50 (0.32, 0.78)	
Risk of death within 12 months, HR (95% CI)^c^				0.013
	65–69 y	3.52 (2.89, 4.27)	2.92 (2.37, 3.59)	
	70–79 y	3.16 (2.77, 3.60)	2.72 (2.38, 3.12)	
	80–89 y	2.29 (1.98, 2.65)	2.07 (1.79, 2.40)	
	90+ y	2.10 (1.61, 2.74)	2.15 (1.63, 2.83)	

Comorbidity^b^				
Outcome		Crude model	Adjusted model^g^	p for interaction^h^
Length of initial hospital admission, HR (95% CI)^c^				0.170
	<4 diag	1.37 (1.27, 1.47)	1.37 (1.27, 1.47)	
	≥4 diag	1.34 (1.26, 1.42)	1.37 (1.29, 1.45)	
Days in hospital one year after nutritional risk screening, RR (95% CI)^d^				<0.001
	<4 diag	1.58 (1.43, 1.73)	1.58 (1.44, 1.74)	
	≥4 diag	1.14 (1.06, 1.22)	1.16 (1.09, 1.24)	
>2 hospital admissions one year after nutritional risk screening, RR (95% CI)^e^				<0.001
	<4 diag	1.58 (1.38, 1.80)	1.58 (1.38, 1.81)	
	≥4 diag	1.02 (0.93, 1.12)	1.04 (0.95, 1.15)	
Risk of death within 12 months, HR (95% CI)^c^				0.001
	<4 diag	3.48 (2.97, 4.08)	3.25 (2.76, 3.83)	
	≥4 diag	2.39 (2.17, 2.63)	2.32 (2.11, 2.56)	

HR = Hazard Ratio, RR = Risk Ratio, Y = years, Diag = diagnoses. The reference group in the models was hospital admissions in which patients were not at risk of malnutrition, except for length of hospital stay (measured as time to hospital discharge), where the HRs were inverted to enable interpretation in the same direction as the other outcomes.

a 65–69 years (n = 2,197), 70–79 years (n = 4,187), 80–89 years (n = 2,819), 90+ years (n = 565).

b <4 diagnoses (n = 3,986), ≥4 diag (n = 5,782).

c Cox regression.

d Negative binomial regression.

e Log-binomial regression.

f Analyses adjusted for sex, prevalence year, number of diagnoses and acute admissions.

g Analyses adjusted for age, sex, prevalence year and acute admissions.

h p for interaction in adjusted model.

Significant interactions were observed between the risk of malnutrition and high comorbidity (defined as ≥4 diagnoses) for the following outcomes: total days in hospital, risk of having more than two hospital stays and risk of death (Table 4). For comorbidity, a risk gradient emerged in the association between the risk of malnutrition and outcomes, with lower risk for outcomes in patients with more than ≥4 diagnoses than in those with <4 diagnoses.

We identified a significant interaction between ACSCs and the risk of malnutrition in terms of the length of initial hospital stay (p for interaction = 0.027, adjusted HR (95% CI) = 1.20 (1.07, 1.37) for the group of patients with an ACSC as the cause of admission versus adjusted HR (95% CI) = 1.32 (1.26, 1.39) for the group of patients with an admission not related to ACSCs). The interaction term for ACSC with the risk of malnutrition was not statistically significant for the other outcomes (see Supplementary Table S4).

No significant interaction was found between sex and risk of malnutrition in any of the study outcomes (see Supplementary Table S4).

### 3.5 The effect of sampling weights

The RR increased by various intervals from 0.10 to 0.35 when adjusting for sampling weights to account for oversampling of long-term stayers in addition to adjusting for age, prevalence year, number of diagnoses and acute admissions in the analyses of associations of risk of malnutrition with total days in hospital and risk of more than two hospital admissions within one year following nutritional risk screening (Supplementary, Table S4).

## 4 Discussion

Our analysis showed that the risks of longer hospital stays, more days in hospital and having more than two hospital admissions within one year following nutritional risk screening were higher in older patients at risk of malnutrition than in patients not at risk of malnutrition. Furthermore, the risk of malnutrition was associated with a two- to threefold higher risk of death within one year.

Our findings of an increased length of initial hospital stay and increased risk of death in older patients at risk of malnutrition are in accordance with previous research reports [4,8]. The review by Bellanti et al. [4] included two studies showing higher 30-day readmission rates among older patients at risk of malnutrition. A recent cohort study also reported a higher risk of readmission within 180 days in older patients at risk of malnutrition [9]. These results are in line with our findings that older patients at risk of malnutrition have a higher risk of having more than two hospital admissions within one year. However, the previous work did not assess the number of days in hospitals during the readmissions [9]. A comprehensive multicentre study that included 73,843 hospital admissions for medical patients found higher odds of death and readmissions in adult patients at risk of malnutrition but did not provide conclusive results regarding length of stay [17]. However, in contrast to the current study, that previous study did not apply survival analysis, allowing for censoring of patients who died before discharge. Thus, the exclusion of patients who died during the hospital stay from their analysis may possibly have underestimated the effect that risk of malnutrition has on the length of hospital stay.

Our results showed that the risk of malnutrition had stronger associations with outcomes in patients 65–79 years old and in patients with fewer than four diagnoses. Two recent studies within the multicentre EFFORT trial by Hersberger et al. (2020) and Efthymiou et al. (2021) also showed similar trends, but with smaller differences between age groups with regard to the associations between being at risk of malnutrition and outcomes [18,19]. The different magnitudes of the findings in our study and their study may be due to different cut-off values applied to categorise patients into age categories (patients at 65–75 and 75+ in the past study versus 65–69, 70–79 and 80+ years in the current study). Another possible explanation may be differences in the study design, as they used an RCT design that requires standardisation while observational studies such as ours allow for a more heterogeneous patient sample [18,19]. This might have led to the finding of a more substantial effect size in our study compared to theirs.

The finding of a more pronounced effect of risk of malnutrition in the ‘65–69’ and ‘70–79’ age groups and in patients with fewer comorbid conditions may be due to several other factors that influence the risk of death and hospitalisation in patients at an advanced age and/or with a high degree of comorbidity. Nevertheless, our findings underline the need for nutritional risk screening and subsequent nutritional support in all groups of older patients, including patients in which malnutrition is not yet evident/observable to healthcare professionals.

To our knowledge, our study is the first large-scale hospital-based study focusing on ACSCs in relation to the risk of malnutrition. Our finding that among patients at risk of malnutrition, the initial hospital admissions more often were due to ACSCs suggests a need for increased attention on possibly preventable hospital admissions in patients at risk of malnutrition. Also, the initial hospital admissions were more frequently acute admissions in patients at risk of malnutrition than in those not at risk. One might speculate that the higher proportion of ACSCs-related and other acute admissions in patients at risk of malnutrition indicates the need for more systematic nutritional practice for older persons in primary care, including better transfer of nutritional information from hospitals to GPs and/or municipality healthcare services upon patient discharge from hospital. Diagnoses are particularly important in this type of communication. In fact, in accordance with Paur et al. [20], we found a very small proportion of patients in this study with diagnoses related to malnutrition. This contrasts with our finding that over one-third of the older patients were at risk of malnutrition according to the nutritional risk screening tool. The lack of transferral of diagnoses related to malnutrition from hospital to primary care might lead to older patients receiving insufficient follow-up and nutritional support after hospital discharge. We also point to the longer length of stay in hospital as an opportunity to initiate both in-hospital nutritional support and collaboration with primary care on further nutritional follow-up for older patients at risk of malnutrition, thereby potentially improving nutritional and clinical outcomes [7,21].

### 4.1 Strengths and limitations

The major strengths of the current study are its large study sample and high participation rate from all somatic wards among the older patients in the included hospitals, as these features increase the generalisability of our findings. Furthermore, the completeness in the point prevalence surveys was high, suggesting a low risk of selection bias. Furthermore, we assessed nutritional risk status using a validated nutritional risk screening tool, the NRS 2002, thereby increasing the study reliability.

Nevertheless, data collection through point prevalence surveys in hospitals might oversample patients with longer hospital stays. The increased length of stay found in the present study, when compared to available national statistics, suggested that this type of length bias may have occurred in our study. We therefore performed analyses with additional adjustments for sampling weights. These analyses showed only small differences in the effect estimates compared to the adjusted model without sampling weights. Furthermore, the sampling weight adjustment led to an increase in the association measure, suggesting that the effect measures obtained from the analyses without adjustment for sampling weights represent conservative estimates of the associations.

One limitation of the present study was that we could not censor patients who died when analysing associations of risk of malnutrition with the total number of days in hospital and the risk of having more than two hospital admissions during the one-year observation period. Consequently, the inclusion of some patients who died might have led to an underestimation of the effect of the risk of malnutrition on these healthcare use outcomes.

The risk of rehospitalisation can also be influenced by numerous factors, such as whether patients have support from informal caregivers, home care services or are nursing home residents. These are all factors that may affect the studied associations, and we cannot rule out any residual confounding due to these other factors.

### 4.2 Conclusion

Malnutrition is a risk factor for rehospitalisation and death among older patients in the year following nutritional risk screening. We found stronger associations of the risk of malnutrition with outcomes in patients in the ‘65–79’ age group and in patients with less comorbidity. This underlines the importance of screening for the risk of malnutrition and the provision of subsequent nutritional support in all groups of older patients, including those entering old age and those with lower disease burden. Future research should aim to investigate the effects of systematic nutritional care, including nutritional support, in different subgroups of older patients and across primary and secondary healthcare settings.

## CRediT authorship contribution statement

KI Folven: Conceptualisation, Data Curation, Formal analysis, Investigation, Writing – Original Draft.

E Biringer: Conceptualisation, Methodology, Formal analysis, Investigation, Writing – Review & Editing, Supervision, Project administration.

RM Nilsen: Methodology, Formal analysis, Investigation, Data Curation, Writing – Review & Editing.

AM Beck: Conceptualisation, Writing – Review & Editing, Supervision.

Kari Sygnestveit: Data Curation, Investigation, Writing – Review & Editing.

Eli Skeie: Data Curation, Writing – Review & Editing.

Ø Hetlevik: Conceptualisation, Writing – Review & Editing.

RJ Tangvik: Conceptualisation, Writing – Review & Editing, Supervision, Project administration.

## Declaration of Generative AI and AI-assisted technologies in the writing process

We did not use generative AI or AI-assisted technologies in the writing process.

## Funding

This work was funded by the Fonna Hospital Trust. The Fonna Hospital Trust had no involvement in the study design, data collection, analysis, writing or decision to submit.

## Declaration of competing interest

All authors declare no conflicts of interest.
