# Supplementary material for: Increased risk of rehospitalisation and death in older hospital patients at risk of malnutrition: A cohort study
Source: J Nutr Health Aging. 2024 Dec 19;29(2):100455. doi: 10.1016/j.jnha.2024.100455 (PMC12180037; doi:10.1016/j.jnha.2024.100455)
Supplement: Supplementary file 1 [file mmc1.docx]

## Supplementary material

**Table S1**

ICD-10 diagnostic codes defined as Ambulatory Care Sensitive Condition (ACSC)

| **Diagnose group** | **ICD-10 codes** |
| --- | --- |
| Dehydration | E86 |
| Constipation | K59.0 |
| Lower respiratory infections | J12, J13, J14, J15, J18, J20, J21, J22 with all underlying codes |
| Urinary tract infection (cystitis) | N30 with all underlying codes except N30.3 and N30.4 |
| Gastroenteritis | A09 with all underlying codes |
| Fractures | S02, S12, S22, S32, S42, S52, S62, S72, S82, S92, T02, T08, T10, T12 with all underlying codes + T14.2 |
| Anaemia | D50, D51, D52, D53 with all underlying codes except D50.1 |
| Pressure ulcers | L89 with all underlying codes |
| Hospital admission due to problems related to care | Z59, Z74, Z75 |

**Table S2**

NRS 2002 screening score for patients in the 4,291 hospital admissions where main screening was performed.

| **NRS 2002 main screening score** | **n, %** |
| --- | --- |
| <3 | 1,135 (26.5) |
| 3 | 1,000 (23.3) |
| 4 | 1,083 (25.2) |
| 5 | 788 (18.4) |
| 6 | 240 (5.6) |
| 7 | 45 (1.1) |

**Table S3**

Risk of death within different time points after nutritional risk screening date

| Outcome | Crude model  HR (95%CI) | Adjusted model^ab^  HR (95%CI) |
| --- | --- | --- |
| Risk of death within 3 months | 3.50 (3.11, 3.93) | 2.86 (2.53, 3.22) |
| Risk of death within 6 months | 3.16 (2.87, 3.47) | 2.63 (2.38, 2.91) |
| Risk of death within 12 months | 2.88 (2.65, 3.13) | 2.45 (2.25, 2.67) |

HR=Hazard Ratio. Reference group in the models was hospital admissions where patients were not at risk of malnutrition ^a^Valid n in

adjusted model = 9,761. ^b^Analyses adjusted for age, sex, prevalence year, no. of diagnoses and acute admissions.

**Table S4**

Risk of health care service use according to nutritional risk status in patients aged ≥65 years stratified by sex, age groups, comorbidity and ambulatory care sensitive conditions (ACSCs), including comparison of models with and without adjustment for sampling weights

**Sex^a^**

| Outcome | Modell | Sex | Crude model | Adjusted model^e^ | Adjusted model with sampling weights |
| --- | --- | --- | --- | --- | --- |
| Length of initial hospital admission (HR (95%CI)) | Cox regression | Both  Male  Female | 1.44 (1.38, 1.51)  1.52 (1.42, 1.62)  1.37 (1.29, 1.47) | 1.31 (1.25, 1.37)  1.35 (1.26, 1.44)  1.27 (1.19, 1.36) | NA |
| Days in hospital one year after nutritional risk screening (RR (95%CI)) | Negative binomial regression | Both  Male  Female | 1.33 (1.25, 1.41)  1.31 (1.21, 1.42)  1.37 (1.25, 1.49) | 1.25 (1.18, 1.32)  1.21 (1.11, 1.32)  1.29 (1.19, 1.40) | 1.38 (1.27, 1.49)  1.32 (1.19, 1.47)  1.45 (1.28, 1.64) |
| >2 hospital stays one year after nutritional risk screening (RR (95% CI)) | Log-binomial regression | Both  Male  Female | 1.22 (1.13, 1.32)  1.17 (1.06, 1.30)  1.30 (1.16, 1.46) | 1.16 (1.06, 1.25)  1.09 (0.97, 1.22)  1.26 (1.11, 1.42) | 1.26 (1.13, 1.41)  1.19 (1.03, 1.37)  1.37 (1.16, 1.62) |
| Risk of death within 12 months (HR (95%CI)) | Cox regression | Both  Male  Female | 2.88 (2.65, 3.13)  2.83 (2.53, 3.16)  3.00 (2.64, 3.39) | 2.43 (2.23, 2.65)  2.41 (2.15, 2.70)  2.51 (2.21, 2.86) | NA |

**Age groups^b^**

| Outcome | Modell | Age groups | Crude model | Adjusted model^f^ | Adjusted model  with sampling weights |
| --- | --- | --- | --- | --- | --- |
| Length of initial hospital admission  (HR (95%CI)) | Cox regression | All  65-69y  70-79y  80-89y  90+y | 1.44 (1.38, 1.51)  1.51 (1.35, 1.69)  1.57 (1.47, 1.69)  1.32 (1.22, 1.44)  1.24 (1.04, 1.48) | 1.29 (1.23, 1.36)  1.24 (1.11, 1.40)  1.38 (1.29, 1.49)  1.25 (1.15, 1.36)  1.20 (1.01, 1.43) | NA |
| Days in hospital one year after nutritional risk screening (RR (95%CI)) | Negative binomial regression | All  65-69y  70-79y  80-89y  90+y | 1.33 (1.25, 1.41)  1.60 (1.40, 1.82)  1.49 (1.36, 1.63)  1.11 (1.01, 1.22)  0.90 (0.74, 1.09) | 1.24 (1.17, 1.32)  1.42 (1.25, 1.62)  1.39 (1,27, 1.52)  1.07 (0.98, 1.17)  0.88 (0.73, 1.06) | 1.38 (1.27, 1.50)  1.77 (1.41, 2.22)  1.56 (1.38, 1.76)  1.16 (1.02, 1.32)  0.77 (0.59, 1.01) |
| >2 hospital stays one year after nutritional risk screening (RR (95% CI)) | Log-binomial regression | All  65-69y  70-79y  80-89y  90+y | 1.22 (1.13, 1.32)  1.33 (1.13, 1.56)  1.44 (1.29, 1.61)  1.09 (0.94, 1.27)  0.48 (0.31, 0.76) | 1.13 (1.04, 1.23)  1.15 (0.97, 1.36)  1.30 (1.15, 1.47)  1.06 (0.91, 1.24)  0.50 (0.32, 0.78) | 1.23 (1.11, 1.37)  1.22 (0.98, 1.51)  1.50 (1.28, 1.77)  1.24 (1.02, 1.52)  0.35 (0.21, 0.61) |
| Risk of death within 12 months (HR (95%CI)) | Cox regression | All  65-69y  70-79y  80-89y  90+y | 2.88 (2.65, 3.13)  3.52 (2.89, 4.27)  3.16 (2.77, 3.60)  2.29 (1.98, 2.65)  2.10 (1.61, 2.74) | 2.49 (2.29, 2.72)  2.92 (2.37, 3.59)  2.72 (2.38, 3.12)  2.07 (1.79, 2.40)  2.15 (1.63, 2.83) | NA |

**(Table S4 continued)**

**Comorbidity^c^**

| Outcome | Modell | Multi-morbidity | Crude model | Adjusted model^g^ | Adjusted model with sampling weights |
| --- | --- | --- | --- | --- | --- |
| Length of initial hospital admission (HR (95%CI)) | Cox regression | All  <4 diag  ≥4 diag | 1.44 (1.38, 1.51)  1.37 (1.27, 1.47)  1.34 (1.26, 1.42) | 1.45 (1.39, 1.52)  1.37 (1.27, 1.47)  1.37 (1.29, 1.45) | NA |
| Days in hospital one year after nutritional risk screening (RR (95%CI)) | Negative binomial regression | All  <4 diag  ≥4 diag | 1.33 (1.25, 1.41)  1.58 (1.43, 1.73)  1.14 (1.06, 1.22) | 1.35 (1.27, 1.43)  1.58 (1.44, 1.74)  1.16 (1.09, 1.24) | 1.47 (1.36, 1.59)  1.77 (1.54, 2.04)  1.20 (1.10, 1.31) |
| >2 hospital stays one year after nutritional risk screening (RR (95% CI)) | Log-binomial regression | All  <4 diag  ≥4 diag | 1.22 (1.13, 1.32)  1.58 (1.38, 1.80)  1.02 (0.93, 1.12) | 1.23 (1.14, 1.33)  1.58 (1.38, 1.81)  1.04 (0.95, 1.15) | 1.35 (1.22, 1.50)  1.74 (1.47, 2.07)  1.07 (0.94, 1.21) |
| Risk of death within 12 months (HR (95%CI)) | Cox regression | All  <4 diag  ≥4 diag | 2.88 (2.65, 3.13)  3.48 (2.97, 4.08)  2.39 (2.17, 2.63) | 2.69 (2.48, 2.93)  3.25 (2.76, 3.83)  2.32 (2.11, 2.56) | NA |

**Ambulatory care sensitive conditions (ACSCs)^d^**

| Outcome | Modell | ACSC | Crude model | Adjusted model^h^ | Adjusted model with sampling weights^*^ |
| --- | --- | --- | --- | --- | --- |
| Length of initial hospital admission (HR (95%CI)) | Cox regression | Both  ACSC  Not ACSC | 1.44 (1.38, 1.51)  1.33 (1.18, 1.50)  1.47 (1.40, 1.54) | 1.31 (1.25, 1.37)  1.20 (1.07, 1.37)  1.32 (1.26, 1.39) | NA |
| Days in hospital one year after nutritional risk screening (RR (95%CI)) | Negative binomial regression | Both  ACSC  Not ACSC | 1.33 (1.25, 1.41)  1.46 (1.29, 1.69)  1.33 (1.25, 1.40) | 1.25 (1.18, 1.32)  1.42 (1.21, 1.66)  1.22 (1.15, 1.30) | 1.38 (1.27, 1.50)  1.54 (1.25, 1.88)  1.36 (1.25, 1.49) |
| >2 hospital stays one year after nutritional risk screening (RR (95% CI)) | Log-binomial regression | Both  ACSC  Not ACSC | 1.22 (1.13, 1.32)  1.41 (1.10, 1.80)  1.21 (1.11, 1.31) | 1.18 (1.08, 1.28)^i^  1.40 (1.09, 1.80)^i^  1.15 (1.06, 1.26)^i^ | 1.28 (1.15, 1.43)^i^  1.45 (1.06, 1.96)^i^  1.27 (1.13, 1.43)^i^ |
| Risk of death within 12 months (HR (95%CI)) | Cox regression | Both  ACSC  Not ACSC | 2.88 (2.65, 3.13)  2.32 (1.83, 2.95)  2.97 (2.72, 3.24) | 2.45 (2.25, 2.67)  2.14 (1.68, 2.73)  2.49 (2.28, 2.73) | NA |

RR=Risk Ratio, Diag=diagnoses. Reference group in the models was hospital admissions in which patients were not at risk of malnutrition except for length of hospital stay (measured as time to hospital discharge), where the HRs were inverted to enable interpretation in the same direction as the other outcomes.

^*^Adjusted for individual sampling weights (i.e. maximum length of stay/individual length of stay) using the *pweight* function in Stata (not available for Cox regression models).

^a^Male (n=4,971), Female (n=4,797)

^b^65-69 years (n=2,197), 70-79 years (n=4,187), 80-89 years (n=2,819), 90+ years (n=565)

^c^<4 diagnoses (n=3,986), ≥4 diag (n=5,782)

^d^ACSC (n=1,125), Not ACSC (n=8,643)

^e^Analyses adjusted for age, prevalence year, no. of diagnoses and acute admissions.

^f^Analyses adjusted for sex, prevalence year, no. of diagnoses and acute admissions.

^g^Analyses adjusted for age, sex, prevalence year and acute admissions.

^h^Analyses adjusted for age, sex, prevalence year, no. of diagnoses and acute admission.

^i^Not adjusted for acute admissions due to multicollinearity in model.
